# Development of Peptic Ulcer following Second Shot of Sputnik V Vaccine: A Case Report and Literature Review of Rare Side Effects of Sputnik V Vaccine

**DOI:** 10.1155/2023/9989515

**Published:** 2023-09-21

**Authors:** Maryam Hasanzarrini, Amir Mohammad Salehi, Samira Nirumandi Jahromi

**Affiliations:** ^1^Clinical Research Development Unit of Shahid Beheshti Hospital, Hamadan University of Medical Science, Hamadan, Iran; ^2^Student Research Committee, Hamadan University of Medical Sciences, Hamadan, School of Medicine, Iran

## Abstract

Considering the global spread of the coronavirus disease 2019 (COVID-19), it is expected that vaccination against its causative agent, the severe acute respiratory syndrome coronavirus 2 (SARS‐CoV‐2), will reduce the related morbidity and mortality. However, the safety of the COVID-19 vaccines and their potential and unknown side effects are a matter of concern. With the ongoing development and implementation of COVID-19 vaccination programs around the world, the side effects, safety, and effectiveness of these vaccines are gradually being reported, providing researchers with valuable information that can affect the production and utilization of the COVID-19 vaccines. The present study intended to report a case of peptic ulcer disease (PUD) development following vaccination with Gam-COVID-Vac, a vector-based COVID‐19 vaccine containing two recombinant human adenoviruses (rAd26 and rAd5).

## 1. Introduction

As a newly emerged disease, the coronavirus disease 2019 (COVID-19) was first reported in Wuhan, China, in December 2019. Having been declared a global epidemic by the World Health Organization (WHO) [1], this disease is associated with considerable mortality and significant social, psychological, and financial impacts globally [2].

Since the outbreak of COVID-19, several treatment protocols have been developed for disease management, with new evidence emerging every day [3]. Moreover, many countries have made efforts to develop effective vaccines to stop the extension of the pandemic. Some of these vaccines have received emergency injection licenses from the WHO, while others are being injected with the permission of local health organizations in different countries [4, 5].

As a COVID-19 vaccine produced by the Gamalia Institute for Epidemiological and Microbiological Research, Sputnik V (Russian: Спутник V) was released on August 11, 2020, and was named the Gam-COVID-Vac (Russian: Гам-КОВИД-Вак) by the Russian Ministry of Health. This vaccine is an adenovirus-based vector vaccine and is injected in two doses with an interval of 21 days [6]. On February 2, 2021, a preliminary analysis of its phase III clinical trial was published in Lancet, reporting an effectiveness of 91.6% with no remarkable side effects [7]. However, this vaccine has not yet been licensed for emergency use by the WHO. Therefore, regarding the studies reporting its effectiveness, ambiguities and objections have been raised [8]. Until February 2021, twenty-one countries had authorized the emergency use of the Sputnik V vaccine [9].

The vaccines against the SARS-CoV-2 have been tested in large randomized controlled trials to evaluate their efficacy and safety in all populations. However, the data from these trials have shown several side effects for each COVID-19 vaccine, which is similar to vaccines against other diseases [4]. Several studies have found that certain groups of people tend to have a lower humoral immune response to vaccines. These groups include older individuals, males, those who are seronegative, and those with underlying health conditions such as end-stage renal disease or diabetes mellitus [10, 11]. As a result, these individuals may require additional doses of vaccination or a combination of different types of vaccines, such as mRNA vaccines with protein vaccines or vector-based vaccines [11]. It is important to note that these groups may have a higher risk of experiencing unknown side effects from vaccination compared to others.

Some of the commonly reported side effects include myalgia, fever, and headache [12]. Moreover, some rare side effects have been reported for these vaccines, mostly the mRNA-based vaccines. The present study reports a case of peptic ulcer disease (PUD) following the second shot of the sputnik V vaccine in a young woman without any underlying disease and reviews the rare side effects reported for this vaccine.

## 2. Case Report

**Figure 1:**
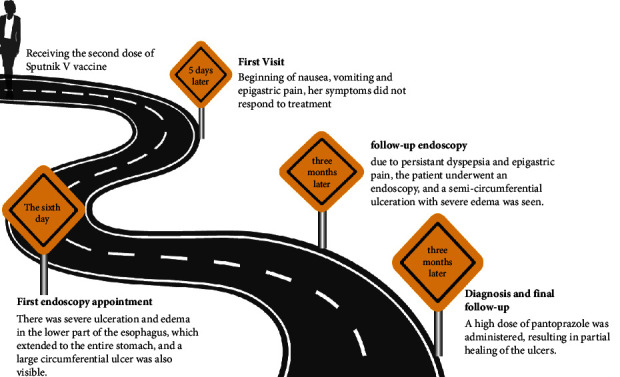
Timeline of the PUD unusual case illustrating the chronological sequence of events.

Our patient was a 28-year-old female healthcare provider who had presented to a physician with nausea, vomiting, and epigastric pain 5 days after being vaccinated with the Sputnik V vaccine. The patient received routine medications (Figure 1). However, her symptoms did not respond to treatment, and she was referred to our healthcare facility for further evaluation.

The patient did not mention any relevant past medical history, underlying diseases, smoking, or alcohol consumption; also, the patient did not mention any special medication or eating habits. Moreover, she had a blood pressure of 120/80 mmHg, a pulse rate of 65 per min, a body temperature of 37°C, a respiratory rate of 22 per min, and no other remarkable finding in her physical examination. Also, she had a high leukocyte count (14,500 per *μ*L, neutrophil percentage: 89%), while other routine tests, including other items of cell blood count (CBC) and differentiation, renal function tests, liver function tests, blood sugar, C-reactive protein (CRP), erythrocyte sedimentation rate (ESR), and urine analysis, were reported in the normal range. Furthermore, she had elevated levels of anti-SARS-CoV-2 antibody, while she underwent two polymerase chain reaction (PCR) tests for SARS-CoV-2, which were reported negative.

**Figure 2:**
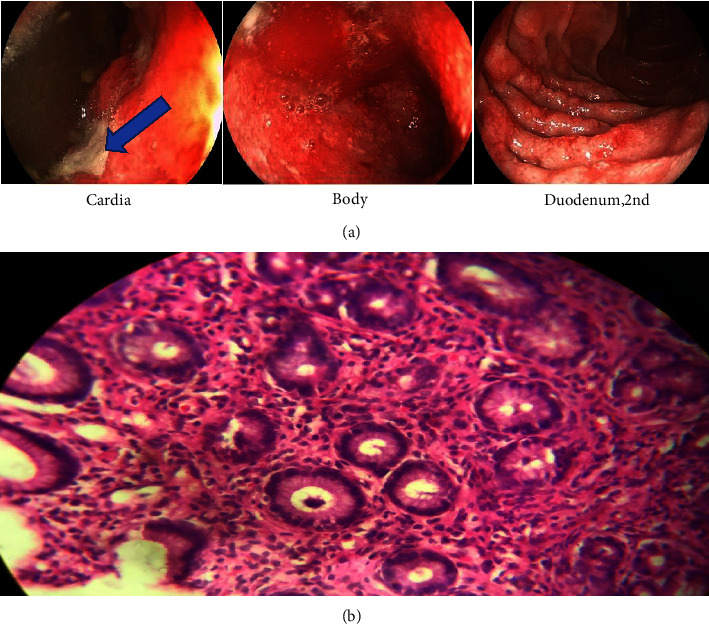
Patient endoscopic findings at the first visit (blue arrow showed PUD) (a) and patient pathology findings at the first visit (b).

Regarding intractable vomiting and severe epigastric pain, the patient underwent an endoscopy, which showed severe ulceration and edema in the lower part of the esophagus extending to the whole stomach. Moreover, a large circumferential ulcer was reported in the gastric body and antrum of which multiple biopsies were taken (Figure 2(a)). Also, she was assessed for gastrin levels, which were reported normal, as the Zollinger–Ellison syndrome was suspected. Furthermore, the pathology reported superficial erosive and ulcerative gastritis, active erosive duodenitis, mild colonization with *Helicobacter pylori*, no dysplasia or neoplasia, and severe inflammation. Moreover, she had leukocyte infiltration in the lamina propria, which was polymorphonuclear-dominant with lower levels of lymphocyte and eosinophil and was associated with red blood cell extravasation and multiple foci of hemorrhage (Figure 2(b)). Finally, the patient was treated with high-dose proton pump inhibitor (PPI, pantoprazole 40 mg QDS) and was discharged after 3-4 days with *H. pylori* eradication medications.

**Figure 3:**
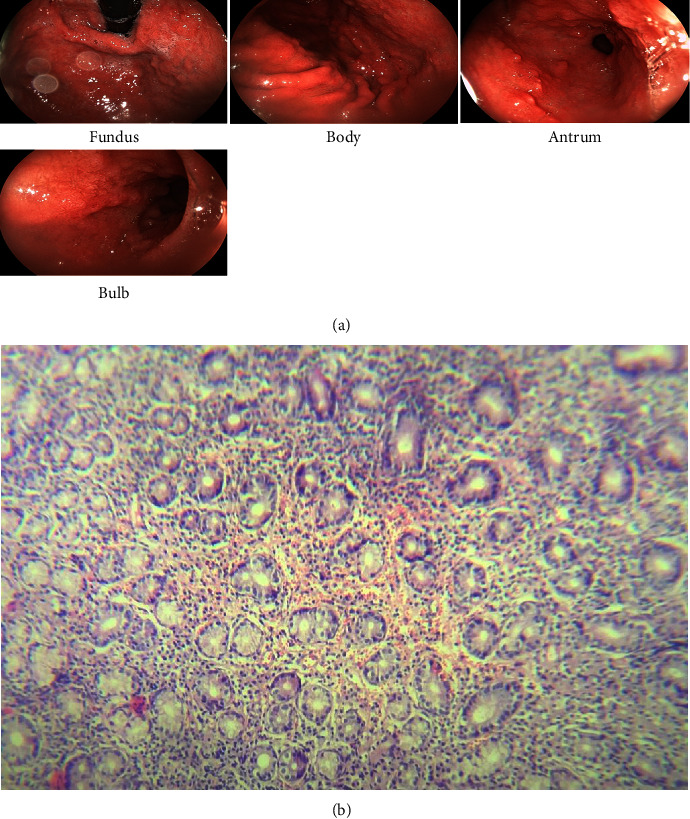
Patient endoscopic findings after 3 months of follow-up (a) and patient pathology findings after 3 months of follow-up (b).

In the following, the patient underwent a follow-up endoscopy three months later due to persistent dyspepsia and epigastric pain. According to the endoscopy report, the esophagus was normal. However, a semicircumferential ulceration with severe edema was reported in the stomach, of which multiple biopsies were taken (Figure 3(a)). Moreover, the pathology reported severe, chronic nonatrophic gastritis with moderate infiltration of lymphocytes and neutrophils in the lamina propria, edema without any dysplasia, neoplastic granuloma, and *H*. *pylori* infection (Figure 3(b)).

Considering the acute onset of the PUD 5 days after the vaccination and no improvement following treatment for *H. pylori* or a family history of PUD, the patient's condition was hypothesized to be a rare side effect of the Sputnik V vaccine. The patient received a high dose of pantoprazole and was advised to skip the third dose of the Sputnik V vaccine. Six months after the first endoscopy, the patient underwent another endoscopy, which revealed partial healing of the ulcers.

## 3. Discussion

Although mass vaccination against COVID-19 is an outstanding achievement, there are several concerns regarding the safety and potential adverse effects of the related vaccines [13]. The common side effects following vaccination against SARS-CoV-2 include injection site reactions, myalgia, headache, fever, and asthenia [13]. However, we reported a confirmed case of PUD 5 days after the second dose of the Sputnik V vaccine.

As a benign mucosal and submucosal lesion of the gastrointestinal tract, PUD is often caused by gastritis, *H. pylori* infection, smoking, and the use of nonsteroidal anti-inflammatory drugs (NSAIDs). Moreover, its rare causes include Zollinger–Ellison syndrome, malignancies, and stress. According to studies, *H. pylori* infection is the main cause of PUD. However, recent studies have shown a relationship between SARS-CoV-2 and peptic ulcers [14, 15]. Also, the symptoms of PUD include epigastric pain, heartburn, indigestion, and blood in the stool, while the pain usually occurs shortly after eating or when hungry [15].

It has been shown that PUD can also occur without *H. pylori* infection. Such condition is referred to as the *H. pylori*-negative PUD, which should be considered if four of the following criteria are met: (i) lack of *H. pylori* bacteria in triple staining of gastric mucosal biopsies (hematoxylin and eosin, Alcian blue stain, and a modified silver stain), (ii) a negative *H*. *pylori* culture, (iii) a negative IgG test for *H. pylori*, and (iv) no self-reported history of receiving treatment for *H. pylori* [16].

In the present study, the patient was diagnosed with *H. pylori* infection and received the related treatment. However, despite the *H. pylori* eradication, the PUD was not completely healed after 3 months. According to the results of the study by Notarte et al., it is shown that 4–6 months after the injection of the second dose of the vaccine, its serum levels decrease, so there is a possibility that the relative healing of our patient's ulcers after 6 months may be due to a decrease in serum levels of vaccine [17].

According to studies on COVID-19 patients with gastrointestinal bleeding (GIB), the virus can be isolated from gastric biopsies. Moreover, the receptors of the angiotensin 2-converting enzyme are expressed in the gastric cells. Therefore, it is possible that SARS-CoV-2 can cause gastritis, PUD, GIB and in rare cases esophagitis dissecans superficialis [18, 19]. However, the underlying mechanisms of vaccine-induced PUD are unclear. A convincing explanation recently suggested for this phenomenon is molecular mimicry [20]. According to this hypothesis, the cross-reaction caused by the similarity of the amino acid sequences of viral antigens and self-antigens may lead to tissue damage by the cytotoxic antibodies [21]. In support of this hypothesis, antibodies against the SARS-CoV-2 spike protein have been reported to show cross-reaction with several human tissue antigens [22]. Moreover, given that our patient developed PUD 5 days after the vaccination, memory T cells must had been sensitized to the antigens similar to SARS-CoV-2 antigens before the vaccination because postvaccination antibody production generally requires several weeks [22].

Our team searched the studies published in PubMed/MEDLINE up to February 22, 2022, to find the case reports of the rare side effects of the Sputnik V vaccine. According to our findings, only 4 related studies were published (Table 1). In all the reported cases, the infection with SARS-CoV-2 was excluded using the real-time PCR (RT-PCR) test, except for one case where the details were not provided [24]. Considering our case report, all the cases were older than 18 years. Moreover, they had a mean age of 37.4 years and included 2 men and 3 women. The developed complications were resolved in all the patients with proper treatment. Also, all the patients had elevated levels of anti-SARS-CoV-2 antibodies after their vaccination, except for one patient with multiple sclerosis (MS) who was treated with rituximab. Despite the acute recurrence of MS in the patient, she was advised to complete her vaccination after the end of her course of treatment with prednisolone (Table 1).

Considering the abovementioned, it is essential for clinicians and researchers to be aware of the potential side effects of COVID-19 vaccination. Moreover, the present study showed the possibility of PUD in patients presenting with nausea, vomiting, and abdominal pain after vaccination with the adenovirus-based COVID-19 vaccines. Given the lack of a history of gastrointestinal problems, it is very likely that our patient developed PUD and gastritis due to vaccination.

## Figures and Tables

**Table 1:** Summary of case reports on Sputnik V vaccines rare side effects.

First authors (references)	Side effect	Sex/age	Symptoms	Physical examination and laboratory findings	Dosage of the vaccine	The time between the onset of symptoms and the injection of the vaccine	Laboratory test performed to exclude other etiologies	Treatment
Naghashzadeh et al. [23]	Lymphocytic myocarditis	Man/29	Chest pain and generalized malaise	Fever tachycardia (110 b.p.m) Hypotension (95/60 mmHg), S3 was noted ST-segment elevation in V3–V4 Troponin 3.04 ng/mL (normal range <0.02), WBC 13 500/mm^3^	2nd	2 day	Serum PCR testings for coxsackievirus, SARS-CoV-2, hepatitis C virus, and human immunodeficiency virus The PCR test on endocardial biopsy for examination SARS-CoV-2, cytomegalovirus, adenovirus, human herpes virus-6 (HHV6), parvovirus B19, enterovirus, and influenza	Methylprednisolone, prednisolone, and mycophenolate mofetil and medical treatment for heart failure: enalapril, carvedilol, and spironolactone

Etemadifar et al. [24]	Acute relapse MS	Female/34	Severe right hemiplegia and ataxia	Muscle force was 2/5 and 3/5 in her right lower and upper limbs knee and biceps reflexes were 2+, bilaterally. No sensory deficits	1st	3 day	NR	Intravenous methylprednisolone (500 mg/day) was administered for five consecutive days

Baimukhamedov et al. [25]	Seropositive rheumatoid arthritis	Female/38	Pain and morning stiffness appeared in the left and right shoulder and small joints of her hands and feet, swelling and pain in both knee joints	Elevated levels of rheumatoid (RA) factor (170 IU/mL), erythrocyte sedimentation (39 mm/h), C-reactive protein (10 mg/L) and anticitrullinated protein antibodies (ACPA) (157 U/mL). DAS28-CRP (6.02). The immunoenzyme SARS-CoV-2 spike IgG antibody test was strongly positive	1st	20 Day	The antinuclear antibody (ANA) screen test, chlamydia and ureaplasma immunoenzyme test, uric acid level (241 mmol/L), serological anti‐SARS‐CoV‐2 rapid test	Methotrexate (15 mg per week), NSAID, and methylprednisolone (100 mg infusion daily for 3 days)

Baimukhamedov et al. [26]	Left elbow joint arthritis	Man/58	Joint swelling, pain, and stiffness upon movement	SARS‐CoV‐2 spike IgG was 2.68 with a positivity coefficient of 13.4	2nd	5 day	SARS‐CoV‐2 PCR, immunoglobulin G (IgG) and IgM antibodies to SARS‐CoV‐2, chlamydia, urea plasma immunoenzyme RA factor, anticyclic citrullinated peptides, and antistreptolysin O levels	NSAID, physiotherapy, and a single intra‐articular injection of diprospan (0.5 mL)

Our case	PUD	Female/28	Nausea, vomiting, and epigastric pain	In endoscopy: severe ulceration and severe edema in the lower part of the esophagus, large circumferential ulcer in the body, and antrum In pathology: severe inflammation, PMN dominant infiltration, less lymphocyte and eosinophil in lamina propria associated with red cell extravasation and multiple foci of hemorrhage	2nd	5 day	SARS‐CoV‐2 PCR, triple staining of gastric mucosal biopsies for *H. pylori*, gastrin level	Pantoprazole 40 mg Q6 hrs

## Data Availability

The data used to support the findings of this study are available on request from the corresponding author.
